# Tailoring focal plane component intensities of polarization singular fields in a tight focusing system

**DOI:** 10.1038/s41598-024-64392-y

**Published:** 2024-06-12

**Authors:** Sushanta Kumar Pal, Leslie A. Rusch

**Affiliations:** Department of Electrical and Computer Engineering, Centre for Optics, Photonics, and Lasers (COPL), Université Laval, Québec, QC G1V 0A6 Canada

**Keywords:** Applied optics, Optical physics, Optical techniques, Imaging and sensing

## Abstract

The scientific community studies tight focusing of radially and azimuthally-polarized vector beams as it is a versatile solution for many applications. We offer a new method to produce tight focusing that ensures a more uniform intensity profile in multiple dimensions, providing a more versatile and stable solution. We manipulate the polarization of the radially and azimuthally polarized vector beams to find an optimal operating point. We examine in detail optical fields whose polarization states lie on the equator of the relevant Poincaré spheres namely, the fundamental Poincaré sphere, the hybrid order Poincaré sphere (HyOPS), and the higher order Poincaré sphere. We find via simulation that the fields falling on these equators have focal plane intensity distributions characterized by a single rotation parameter $\alpha$ determining the individual state of polarization. The strengths of the component field distributions vary with $\alpha$ and can be tuned to achieve equal strengths of longitudinal (*z*) and transverse (*x* and *y*) components at the focal plane. Without control of this parameter (e.g., using $\alpha =0$ in radially and $\alpha =\pi$ in azimuthally-polarized vector beams) intensity in *x* and *y* components are at 20% of the *z* component. In our solution with $\alpha =\pi /2$, all components are at 80% of the maximum possible intensity of *z*. In examining the impact of $\alpha$ on a tightly focused beam, we also found that a helicity inversion of HyOPS beams causes a rotation of 180 degree in the axial intensity distribution.

## Introduction

By manipulating the polarization degree of freedom we can control the properties of light beams. Beams with spatially uniform polarization across the transverse plane have typically been exploited. Polarization and related spin properties are important characteristics of electromagnetic waves and their manipulation is crucial in almost all photonic applications. In recent years, beams with spatially varying polarization distributions across the transverse plane have been exploited for several applications. Among these, polarization singular beams have been of particular interest for optical communication^1,2^, optical signal processing^3^, optical chirality measurements^4^, optical trapping and manipulation^5^, optical lattices^6,7^, material machining^8^, and structured illumination microscopy^9^.

For polarization singular beams (PSBs) with spatially varying distributions, we can classify them as either hybrid order Poincaré sphere (HyOPS) or higher order Poincaré sphere (HOPS) beams. In a tight focusing optical system the HOPS beams are known to produce smaller focal spots compared to the linearly polarized light^10–13^. The tight focusing of HOPS and HyOPS beams produces significant longitudinal field components^11,13–17^ and these components are used for the generation of complex 3D polarization structures namely optical Möbius strips^18–28^, twisted ribbons^29^, and optical cones^18^. The additional longitudinal field component paves the way to realize 3D polarization structures and singularities^30,31^.

In the past tight focusing of specific HOPS singularities (V-points) such as radially and azimuthally polarized fields have been used to shape the focal spot intensity distribution. In the case of HyOPS singularities (C-points), the tight focusing of generic singularities such as lemon and star-type C-point singularities have been used to realize three-dimensional optical polarization Möbius strips and shape the focal plane intensity distributions. Owing to the emerging interest and vast applications of these beams, there is a need for a complete study of the degenerate polarization singularity index states. We study the modulation of the focal plane not just $|E|^2$ but each component’s focal plane intensity distributions for the degenerate polarization singularity index states of HOPS and HyOPS. The current study will help the scientific community better understand polarization singular beams.

## Spatially inhomogeneous polarization singular beams

The singularities of HyOPS beams are known as C-points and L-lines^32–34^. The singularities of HOPS beams are known as V-points^33,35^. The C-points are the points of circular polarizations surrounded by spatially varying elliptical polarization states; at C-points the orientation of the polarization ellipse is undefined. The L-lines are points of linear polarizations in the spatially varying elliptical polarization states; at L-lines the polarization handedness of the ellipse is undefined. Unlike C-points and L-lines, the V-points are the points of intensity nulls in the spatially varying linearly polarized states.

The C-point or V-point index is given by

1 $$\begin{aligned} \frac{1}{2\pi } \oint \overrightarrow{\nabla } \gamma \cdot \overrightarrow{dl}, \end{aligned}$$

where $\gamma$ refers to the azimuth of the polarization ellipse. For the V-point, $\gamma$ will orient the linear polarization states around the singular point. The V-point index $\eta$ can acquire only integer values. For the C-point, $\gamma$ will be the orientation of the major axis of the polarization ellipses around the singular point. The C-point index is referred to as $I_C$, and the V-point index is known as the Poincaré–Hopf index ($\eta$)^33,34,36^. The C-point index $I_C$ can acquire both integer and half-integer values. Unlike V-points, the C-points are associated with an additional parameter namely helicity^37,38^. A C-point can be either left or right helicity.

The singularities of HOPS (V-points) and HyOPS (C-points) beams can be realized by the superposition of right and left circular polarization states with appropriate orbital angular momentum. For a V-point the circular polarization states carry equal and opposite orbital angular momentum, leading to the intensity null at the singularity point. Unlike V-points, a C-point singularity can have any intensity. Based on the intensity value at the singular point, a C-point is called a bright or dark C-point^32,39^. For a bright C-point singularity, one of the two circular polarization states has a plane wavefront (i.e., zero orbital angular momentum). For a dark C-point singularity, both the circular polarization states carry nonzero and unequal orbital angular momentum. For our study, we only consider bright C-points as they are easier to generate experimentally and therefore in more common usage.

An optical field embedded with a polarization singularity can be decomposed into a circular polarization basis with right circular, $\hat{e}_{R}$, and left circular, $\hat{e}_{L}$, unit basis vectors. Let the integers $l_1$, and $l_2$ be the orbital angular momentum (OAM) states of the phase vortex beams with amplitude scaling factors $A_1$ and $A_2$ respectively. An optical field with a polarization singularity can be expressed as^34^,

2 $$\begin{aligned} \textbf{E}(r,\theta )&= e^{\frac{-r^2}{w_0^2}} \left[ A_1 r^{\mid l_1 \mid } e^{il_1 \theta } \hat{e}_R+ A_2 r^{\mid l_2 \mid }e^{i(l_2 \theta + \alpha )} \hat{e}_L\right] , \end{aligned}$$

where *r*, $\theta$, and $w_0$ are the radial distance, azimuthal angle, and waist radius of the polarization singular beam, respectively. The parameter $\alpha$ is the rotation angle that sweeps the degenerate states of a given polarization singularity index.

**Figure 1:**
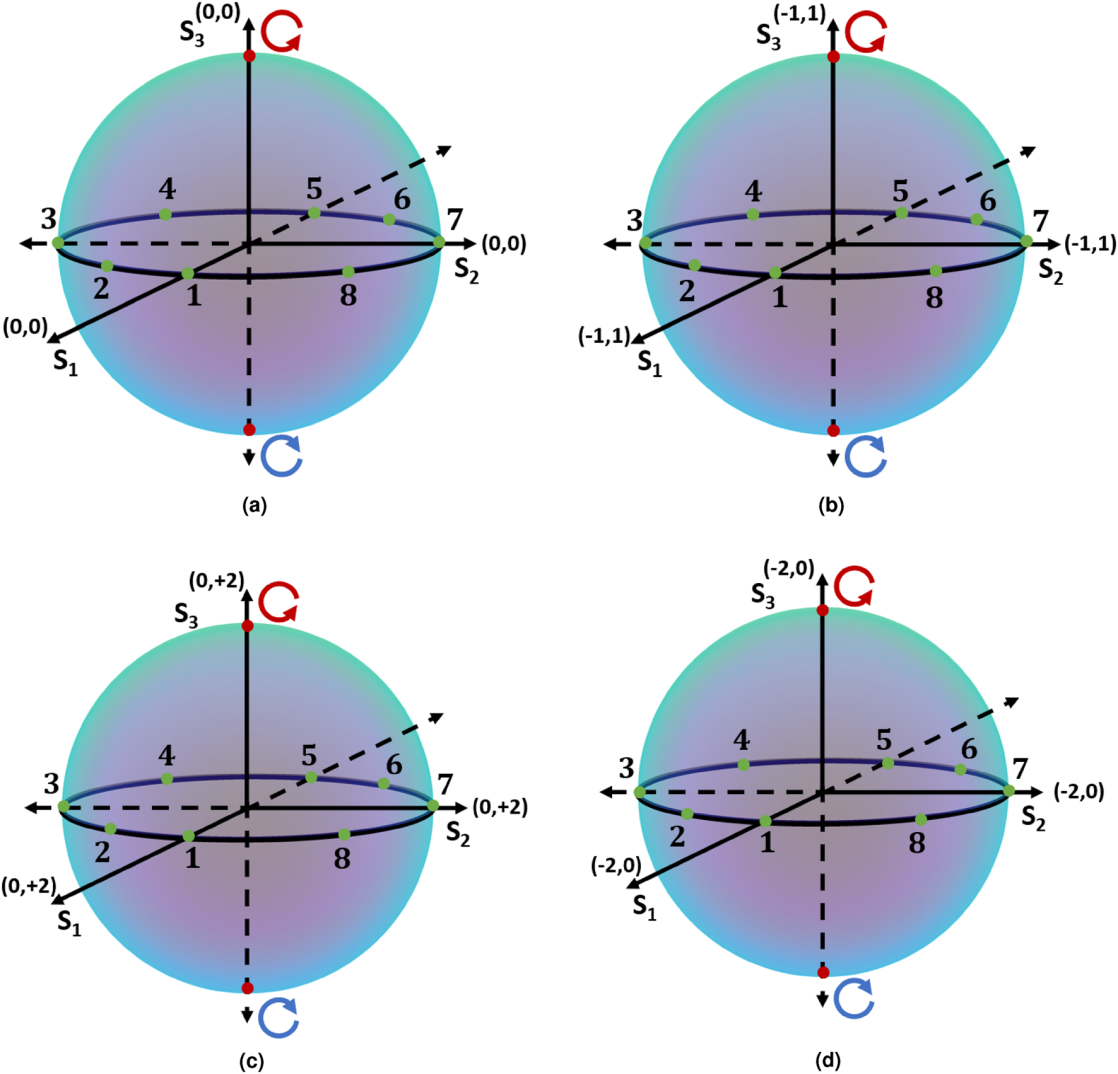
Schematic diagram of circular basis FPS, HOPS, and HyOPS. In each sphere, the superscripts in the coordinate axes $S_1$, $S_2$, and $S_3$ denote the content of OAM in the right ($\hat{e}_R$) and left ($\hat{e}_L$) circular polarization eigenstates, respectively.

The use of Poincaré spheres^40,41^ has greatly simplified the understanding of geometric phases in polarization optics. The normalized Stokes parameters $S_1$, $S_2$, and $S_3$ are the coordinates on the surface of a Poincaré sphere (PS); each corresponds to a unique polarization state. The north and south poles of a Poincaré sphere have right and left circular polarization, respectively. An alternate basis for the Poincaré sphere uses ($2\gamma$, $2\chi$). Here $\gamma$ is azimuth and $\chi$ is the ellipticity of the polarization state, found from $2\gamma =\tan ^{-1} \left( {S_2}/{S_1}\right)$ and $2\chi =\sin ^{-1} \left( {S_3}/{S_0}\right)$. The parameters $A_1$ and $A_2$ in Eq. (2) can be tuned to control the ellipticity of the polarization ellipse, whereas the azimuth of the polarization ellipse can be controlled by tuning the parameter $\alpha$.

Many versions of the PS exist to represent different types of beams. The schematic of four different versions of Poincaré spheres are depicted in Fig. 1. The fundamental Poincaré sphere (FPS) is used for conventional polarization states, namely linear polarization, circular polarization, and elliptical polarization. An FPS is shown in Fig. 1a. Optical fields represented on an FPS can be realized from Eq. (2) with $l_1=0$, $l_2 = 0$; $A_1$ and $A_2$ specify various polarization states. Here, the eigenstates are right circularly polarized (RCP) and left circularly polarized (LCP) plane beams, marked by red points on the sphere.

Polarization singular beams with spatially varying distributions are represented on the hybrid order Poincaré sphere (HyOPS)^42^ or the higher order Poincaré sphere (HOPS)^43^. On the HOPS the V-point singularities are points on the equator. The HOPS beams can be realized from Eq. (2) by putting $A_1=A_2=1$, and $l_1=-l_2$ for different orders of HOPS. Figure 1b depicts a HOPS in a circular basis for V-point singularities with index $\eta =+1$. Here, the eigenstates are RCP and LCP vortex beams of topological charges $l_1=-1$, and $l_2=+1$, respectively, marked by red points on the sphere.

A C-point singular beam can be derived from Eq. (2) when $l_1 \ne l_2$ and unique values of $A_1$ and $A_2$. As we are only interested in bright C-point singularities, either $l_1$ or $l_2$ are zero. Since C-point singularities can have one of two helicities, we need two HyOPS to represent C-point singularities of the same polarization singularity index. Figure 1c depicts one HyOPS in a circular basis for bright C-point singularities with index $I_C=+1$. Here, the eigenstates are the RCP plane beam and LCP vortex beam of topological charges $l_1=0$, and $l_2=+2$, respectively, marked by red points on the sphere. For the second helicity, we have another HyOPS in Fig. 1d; note the change in axis definition. Here the eigenstates have helicity that is inverted relative to those in Fig. 1c, with the RCP vortex beam and LCP plane beam of topological charges $l_1=-2$, and $l_2=0$, respectively, marked by red points on the sphere.

## Theory of the tight focusing

Scalar diffraction theory is widely used to explain several physical phenomena in the paraxial approximation condition. In a high-numerical aperture optical system, the scalar diffraction theory fails to explain the tight focusing behavior of the optical field. In the high numerical aperture optical system, Richards-Wolf vector diffraction integral theory is used instead of scalar-diffraction theory to calculate the field distributions^44^. Let us consider an optical system with azimuthal angle and focusing angles denoted by $\theta$ and $\phi$ respectively. The maximum focusing angle or maximum angle of convergence is given by $\phi _{max}$ (we consider $\phi _{max} = 75^\circ$). We consider the optical fields with amplitude distributions, $E_2 (\phi )$, at the input plane of a high numerical aperture lens given by

3 $$\begin{aligned} E_2 (\phi )= E_0 e^{(-\beta ^2 \sin ^2 \phi / \sin ^2 \phi _{max})}, \end{aligned}$$

where $E_0$ is the maximum amplitude of the input beam. The parameter $\beta$ is called the truncation parameter and is given by $\beta =a/w_0$, where *a* is the radius of the lens and $w_0$ is the beam waist. The numerical aperture (NA) of an optical system with refractive index *n* and maximum focusing angle $\phi _{max}$, can be expressed as $NA=n \sin \phi _{max}$. For an aplanatic lens system, the apodization factor can be written as $A_3(\phi ) = \sqrt{\cos \phi }$.

The polarization distribution of the input polarization singular beam at the exit pupil of the high numerical aperture lens system can be expressed as

4 $$\begin{aligned} \begin{aligned} \textbf{P}(\phi ,\theta )= \left[ \begin{matrix} a_1 (\cos \phi \cos ^2 \theta + \sin ^2 \theta ) + b_1 (\cos \phi \sin \theta \cos \theta - \sin \theta \cos \theta ) \\ a_1 (\cos \phi \sin \theta \cos \theta - \sin \theta \cos \theta ) + b_1 (\cos \phi \sin ^2 \theta + \cos ^2 \theta ) \\ -a_1 \sin \phi \cos \theta - b_1 \sin \phi \sin \theta \end{matrix} \right] , \end{aligned} \end{aligned}$$

where the amplitudes of the *x* and *y* components of the input polarization singular beam are given by the parameters $a_1(r,\theta )=(r^{|l_1|} e^{il_1\theta } + r^{|l2|} e^{i(l_2\theta +\alpha )})$ and $b_1 (r,\theta )=i(r^{|l_1|} e^{il_1\theta } - r^{|l_2|} e^{i(l_2\theta +\alpha )})$, respectively. The focal plane coordinates (*u* and *v*) are given by

5 $$\begin{aligned} u&=k z_P \sin ^2\phi _{max} \\ v&=k \sin \phi _{max} \sqrt{x_P ^2 + y_P ^2} ,\end{aligned}$$

where $\lambda$ is the wavelength of the optical field and *k* is the propagation vector and is given by $k=2\pi / \lambda$. The $x_P$, $y_P$, and $z_P$ are the Cartesian coordinates of a point in the observation plane. The electric field in the focused region of polarization singular beam passing through an aberration-free high numerical aperture lens is expressed as^44,45^

6 $$\begin{aligned} \textbf{E}(u,v)= & {} \left[ \begin{matrix} E_x(u,v)\\ E_y(u,v)\\ E_z(u,v) \end{matrix}\right] = \left( -\frac{iA_0}{\lambda }\right) \int _{0}^{\phi _{max}}\int _{0}^{2\pi } E_2(\phi ) \textbf{P}(\phi ,\theta ) A_3(\phi ) e^{(-i \frac{v}{\sin \phi _{max}} \sin \phi \cos \left( \theta - \theta _P)\right) }\nonumber \\ {}{} & {} \times e^{\left( -i \frac{u}{\sin ^2 \phi _{max}} \cos \phi \right) } \sin \phi d\phi d\theta , \end{aligned}$$

where parameter $A_0$ is linked to the optical system, $\theta _P$ is the azimuthal position coordinate of a point in the observation plane. and the integral is known as the Richards-Wolf integral.

**Figure 2:**
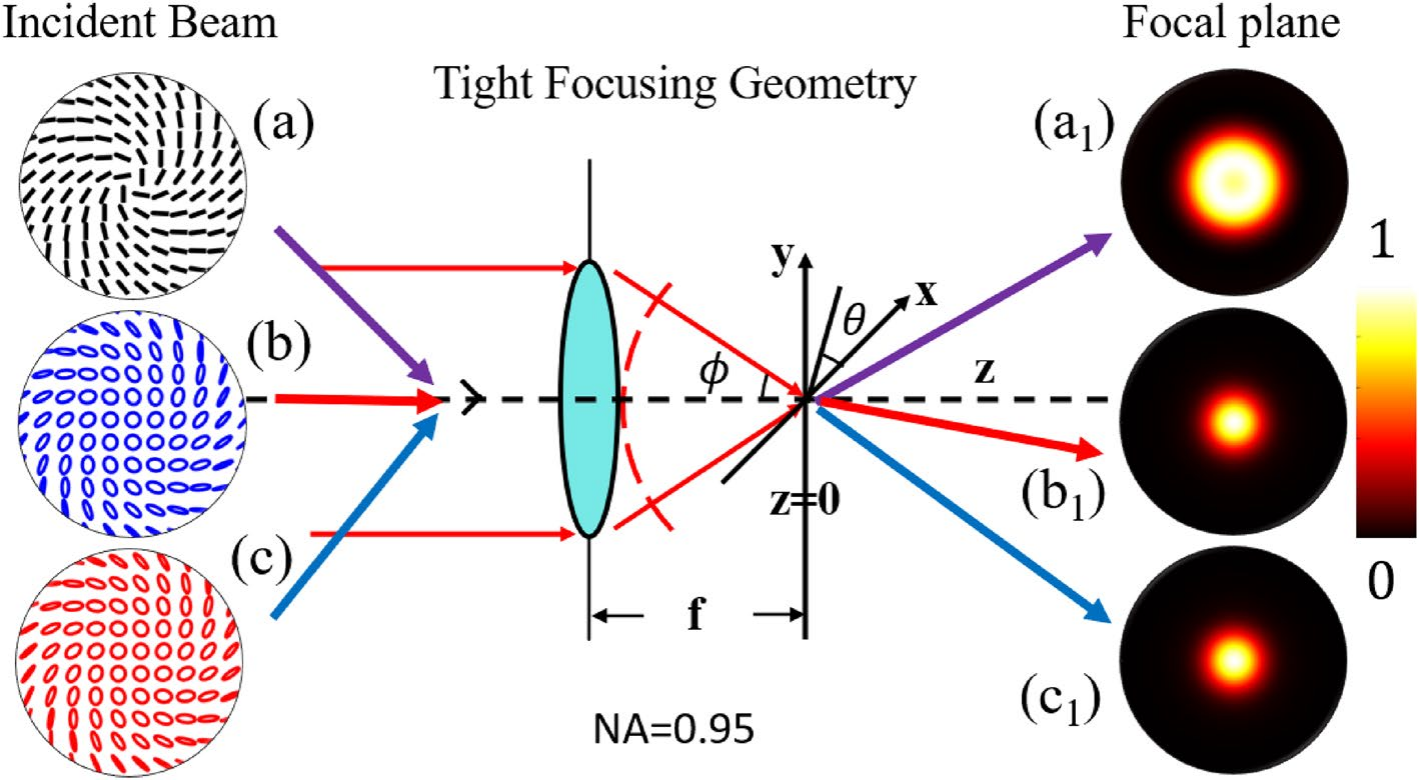
Schematic diagram of the tightly focused optical system.

We consider a high numerical aperture lens (NA$=0.95$) system, as shown in Fig. 2, to study the focal plane field distributions of various optical fields. We show three classes of polarization singular fields with different transverse plane polarization distributions as the inputs labeled (a) HOPS beam, (b) HyOPS beam with right helicity, and (c) HyOPS beam with left helicity. The input beam is tightly focused in simulation using Eq. (6). We solved Eq. (6) numerically in Matlab^TM^ to calculate field distributions at the focal plane of the high numerical aperture lens. The corresponding output focal plane intensity distributions are labeled (a_1_)–(c_1_).

**Figure 3:**
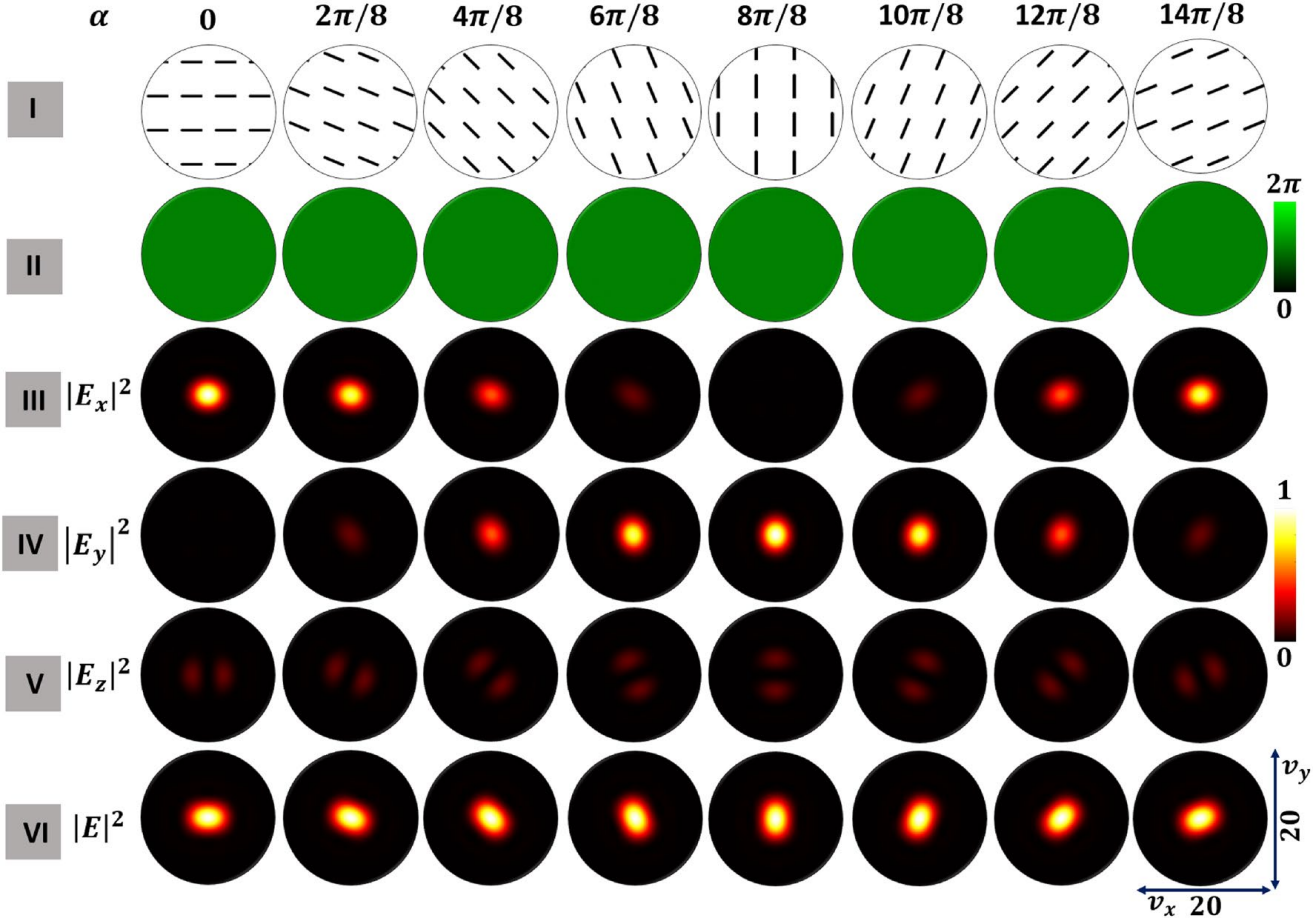
For a FPS beam, focal plane distributions of tightly-focused optical fields for various $\alpha$; rows show incident beam spatial distributions of (I) polarization, (II) $S_{12}$-Stokes field phase, and the constituent focal plane intensity (III) $|E_x|^2$, (IV) $|E_y|^2$, and (V) $|E_z|^2$, and (VI) total intensity $|E|^2$.

**Figure 4:**
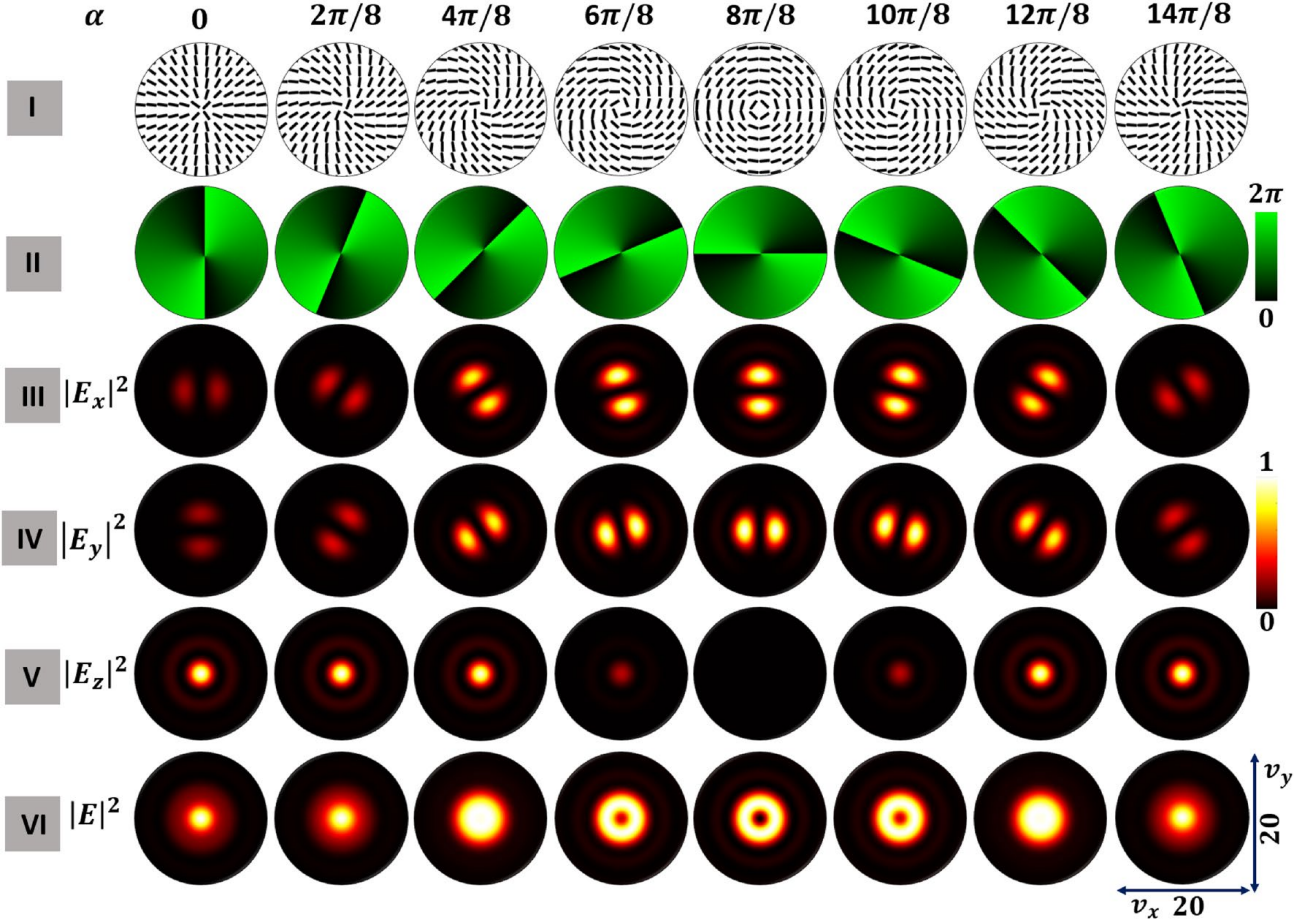
For a HOPS beam of Poincaré–Hopf index $\eta =+1$, focal plane distributions of tightly-focused optical fields for various $\alpha$; rows show incident beam spatial distributions of (I) polarization, (II) $S_{12}$-Stokes field phase, and the constituent focal plane intensity (III) $|E_x|^2$, (IV) $|E_y|^2$, and (V) $|E_z|^2$, and (VI) total intensity $|E|^2$.

**Figure 5:**
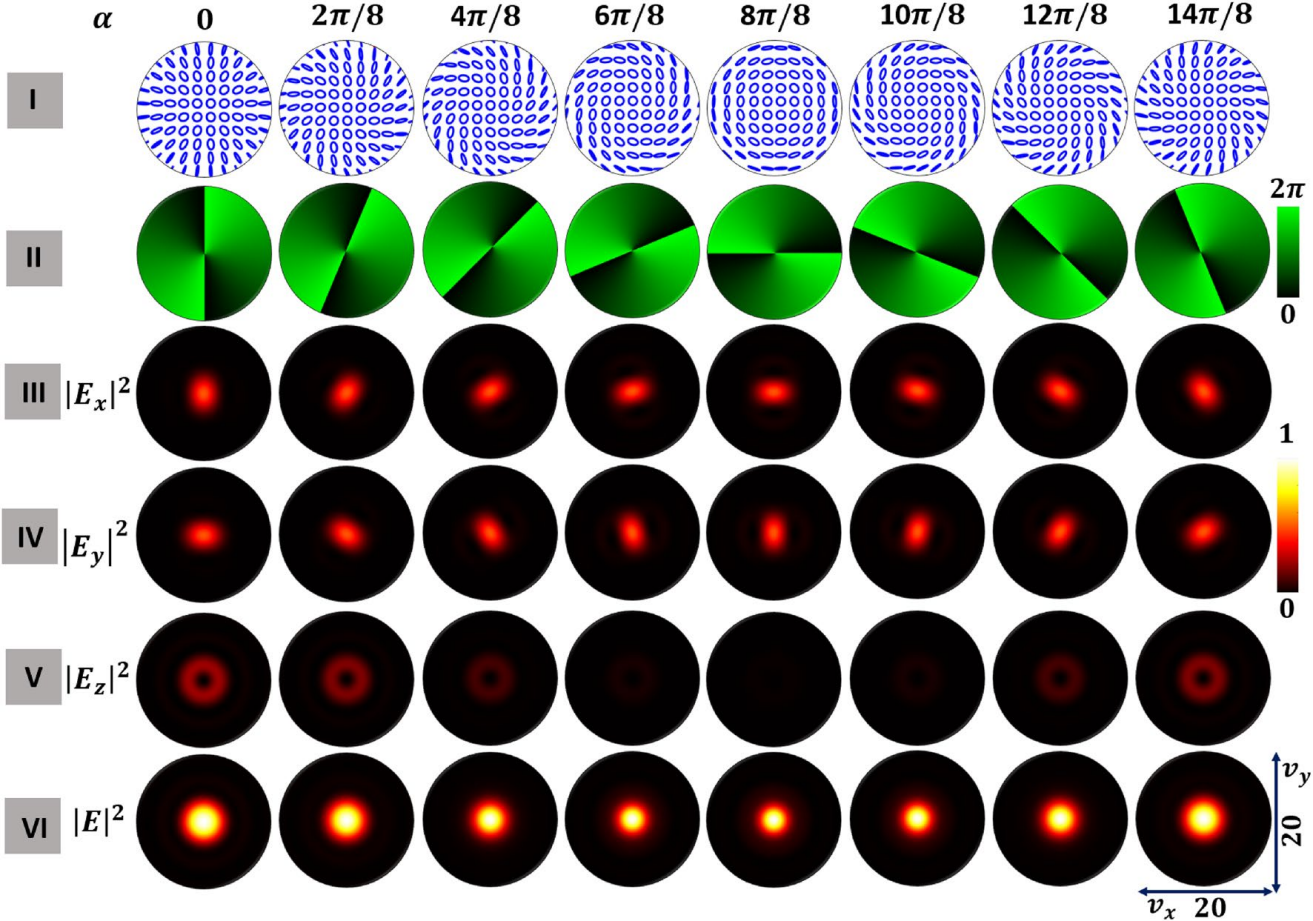
For a right-handed HyOPS beam of polarization singularity index $I_C=+1$, focal plane distributions of tightly-focused optical fields for various $\alpha$; rows show incident beam spatial distributions of (I) polarization, (II) $S_{12}$-Stokes field phase, and the constituent focal plane intensity (III) $|E_x|^2$, (IV) $|E_y|^2$, and (V) $|E_z|^2$, and (VI) total intensity $|E|^2$.

## Tight focusing of degenerate states of polarization singular beams

The parameter $\alpha$ in Eq. (2) is the rotation angle that sweeps the degenerate states of a given polarization singularity index, varying between 0 and $2\pi$. All polarization states with $0 \le \alpha < 2\pi$ have unique polarization distributions, so we restrict our attention to this range. We consider eight values of $\alpha$ and compute the normalized Stokes parameters of different polarization singular beams for each $\alpha$. We plot the resultant degenerate polarization states on the equatorial plane of the appropriate Poincaré sphere in Fig. 1; label 1 refers to $\alpha =0$, up to label 8 for $\alpha =14\pi /8$.

We compute the spatial distributions of polarization and intensity for each $\alpha$ with the following methodology. Using the respective circular components of the polarization singular beams from Eq. (2), we normalize the Stokes parameters to compute the azimuth $\gamma$ and ellipticity $\chi$ spatial distributions. We use these two parameters successively to find the transverse plane polarization distributions of the respective optical fields. The $S_{12}$ Stokes field phase distribution is given by $\phi _{12}=tan^{-1} \left( {S_2}/{S_1}\right)$. The polarization singularities will appear as discontinuities in the $S_{12}$-Stokes field phase distribution. If the $S_{12}$ Stokes field phase distributions are spatially inhomogeneous, they will vary with $\left( x, y\right)$. For simplicity, the intensity profiles of all the optical fields chosen for tight focusing are considered to have a Gaussian distribution. For each polarization singular beam, we evaluated the Richards-Wolf integral (Eq. 6) to find the focal plane intensity distributions for transverse components (*x* and *y*) and longitudinal components (*z*). We present results for FPS in Fig. 3, HOPS in Fig. 4, and right-handed HyOPS in Fig. 5.

In Fig. 3 we begin with the FPS beams. The first row (I) presents the transverse plane polarization distributions for eight distinct $\alpha$. The $S_{12}$-Stokes field phase distributions of these fields are presented in the second row (II). Since the polarization distributions are spatially homogeneous, the $S_{12}$-Stokes field phase distributions are uniform across the transverse plane. The constituent focal plane intensity distributions $|E_x|^2$ (*x*-component), $|E_y|^2$ (*y*-component), and $|E_z|^2$ (*z*-component) are shown in rows III, IV, and V, respectively. As $\alpha$ increases from 0 to $\pi$, the strength of the *x*-component intensity distribution decreases from maximum to minimum and then increases again as $\alpha$ increases from $\pi$ to $2\pi$. The strength of the *y*-component intensity distribution is out-of-phase with the strength of the *x*-component; when $|E_x|^2$ waxes, $|E_y|^2$ wanes and vice-versa. While $|E_z|^2$ rotates with $\alpha$, the total intensity of the longitudinal component (*z*-component) distribution remains constant with $\alpha$. We calculate the focal plane collective intensity distributions $|E|^2$ from the respective transverse and longitudinal components intensity distributions and present them in row VI. As with $|E_z|^2$, the collective intensity rotates with $\alpha$, while the strength of the collective intensity remains constant with $\alpha$.

In Fig. 4 we present results for $\eta =+1$ HOPS beams. The transverse plane polarization distributions are spatially inhomogeneous, as seen in the first row (I). As a result, the $S_{12}$-Stokes field phase distributions of these fields are spatially varying as seen in row (II). In the case of HOPS beams, for a given $\alpha$, distributions for both *x* and *y* components have the same total strength. The strengths of both transverse component distributions grow until $\pi$ and then decrease. The distributions rotate with $\alpha$, but the relative rotation of *x* and *y* components remains constant. This rotation also occurs in Fig. 3 for FPS beams but is not visible due to the circular symmetry of those distributions. Unlike FPS beams, for the HOPS beams the strength of the longitudinal component varies with $\alpha$ and is circularly symmetric.

Figure 5 presents results for right-handed $I_C=+1$ HyOPS beams. The transverse plane polarization distributions are shown in row (I), and the $S_{12}$-Stokes field phase distributions in row (II). For HyOPS beams, the strengths of the transverse intensity distributions remain constant with $\alpha$; rotation of the component distributions is as seen in Fig. 4. The strength of the longitudinal component varies with $\alpha$, with a period of $2\pi$. It follows that the collective intensity distribution tracks the behavior in the longitudinal component, i.e., varying with $\alpha$ with a period of $2\pi$. While not shown, similar results were found for the left-handed $I_C=+1$ HyOPS beams.

## Results

### Tuning to achieve uniform maximal intensity

**Figure 6:**
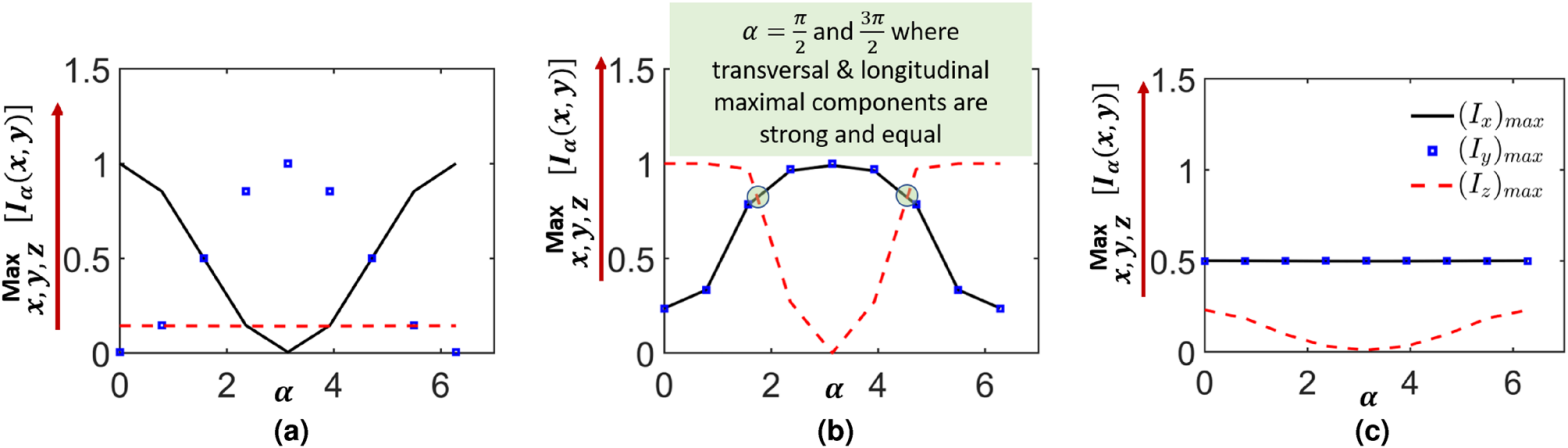
Variations of maximum intensity values of $|E_x|^2$, $|E_y|^2$, and $|E_z|^2$ with $\alpha$ for (**a**) Linearly polarized beams (FPS Beams); (**b**) HOPS beams with $\eta =+1$; (**c**) left- or right-handed HyOPS beams with $I_C=+1$.

We have examined how the strengths of the component field distributions at the focal plane depend on the parameter $\alpha$. We explore the intensity modulation $I_{\delta }\left( v_x,v_y \right)$ with $\alpha$, where $\delta =x,y,z$ reported in Figs. 3, 4 and 5. We plot in Fig. 6 the maximum value over $v_x,v_y$ of the strengths of the constituent focal plane intensity distributions. We present results for FPS in Fig. 6a, HOPS in Fig. 6b, and right-handed HyOPS in Fig. 6c, for $\alpha$ varying from 0 to $2\pi$. For FPS beams both *x* and *y*-component maximum intensities have a periodicity of $2\pi$ with $\alpha$, whereas for HOPS beams all three components have a periodicity of $2\pi$. For the left- or right-handed HyOPS beams, only the maximum of the longitudinal component (*z*-component) shows a periodicity of $2\pi$.

In the case of FPS beams, the maximum strengths of *x* and *y*-components vary sinusoidally between $0-1$ and $1-0$ with $\alpha$ respectively, whereas the maximum value corresponding to the *z*-component remains constant for all values of $\alpha$. For HOPS beams, the change in $\alpha$ leads to the simultaneous modulations of strengths of all three component intensity distributions. The maximum intensity of *x* and *y*-components varies between 0.4 and 1 with $\alpha$ similarly. In contrast, the maximum intensity of the *z*-component varies between 1 and 0 with $\alpha$. For right-handed HyOPS beams, the maximum intensity of the *x* and *y*-components remain constant with $\alpha$, whereas the maximum intensity of the *z*-component varies between 0.3 and 0 with $\alpha$. Similar variations were observed for the left-handed HyOPS beams.

Among the three types of beams we examine (FPS, HyOPS, and HOPS), the HOPS beams are unique in their ability to generate both non-negligible and equal-strength longitudinal and transverse components at the focal plane of a high numerical aperture optical system. Not all the HOPS beams have this important feature. We have shown the HOPS beams with unique superposition of radial and azimuthal polarizations demonstrate this feature. These specific HOPS beams can be realized by setting $\alpha = n\pi /2$ in Eq. (2), where *n* is an odd integer. This is not possible for FPS and HyOPS beams.

To the best of our knowledge, this feature of HOPS beams has not been shown to date. The ability to tailor the strengths of constituent field distributions within the focal volume for different Poincaré sphere beams will be of significant interest for a multitude of advanced applications in the field of microstructuring of polarization-sensitive materials^46,47^, optical micromanipulations^48,49^, and materials micro magnetization^50,51^.

### Axial intensity distribution dependence on input beam

**Figure 7:**
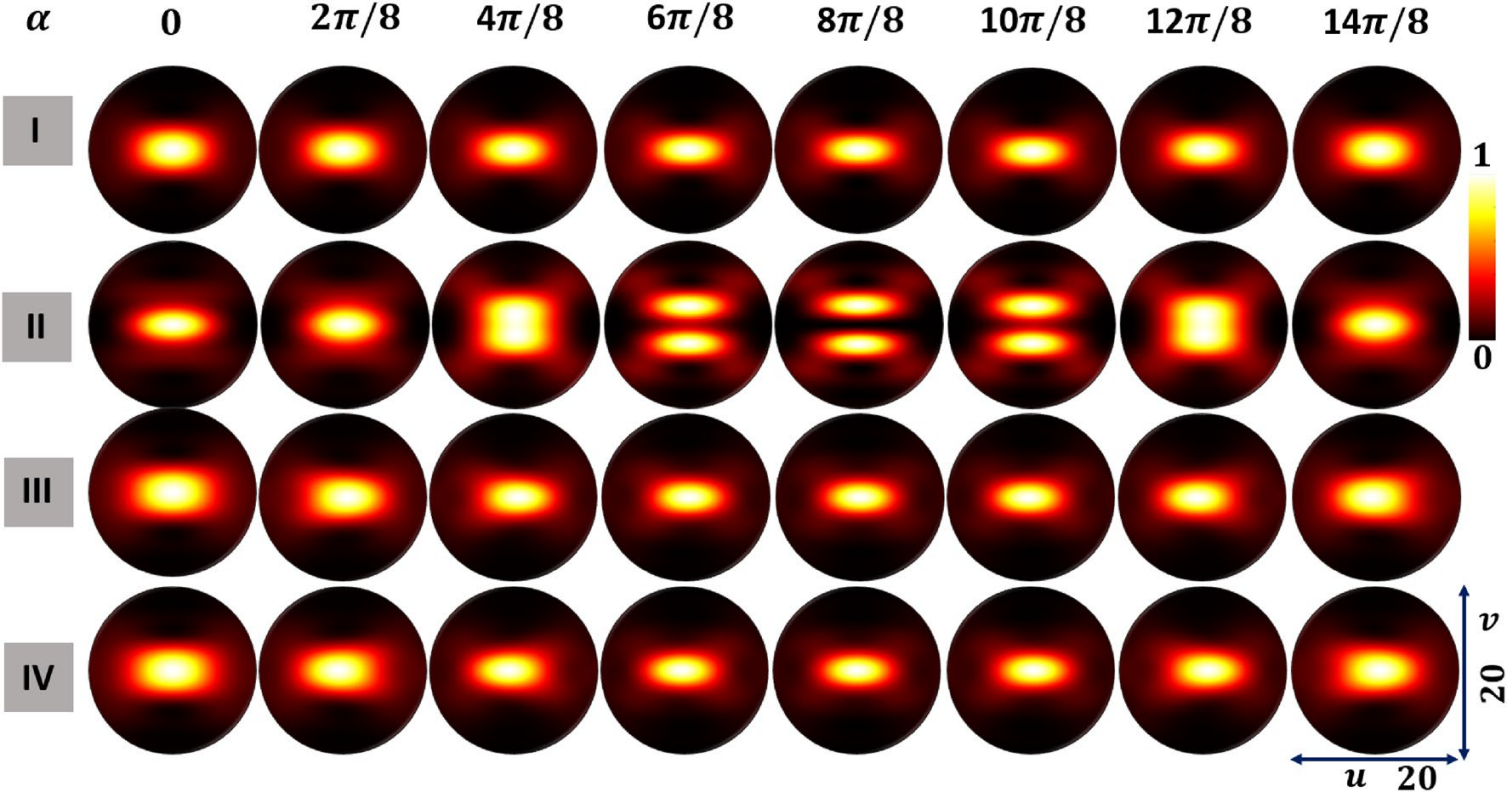
Total intensity distributions in the *xz*-plane of a lens with $NA=75^\circ$ for optical fields with various $\alpha$; rows show axial plane intensity distributions of (I) FPS beams, (II) HOPS beams with $\eta =+1$, (III) right-handed HyOPS beams with $I_C=+1$, and (IV) left-handed HyOPS beams with $I_C=+1$.

For a tightly focused beam, we also examined the impact of $\alpha$ on the axial intensity distribution. Figure 7 shows the total intensity distributions in the $x-z$ plane of the focal region of an aberration-free aplanatic focusing system with $NA=0.95$. The axial intensity distributions for FPS beams for different values of $\alpha$ are presented in row I of Fig. 7. For the FPS beams the width of the axial intensity distributions along the vertical direction reduces symmetrically as $\alpha$ varies from 0 to $\pi$ and then increases as $\alpha$ varies from $\pi$ to $2\pi$. Row II of Fig. 7 shows the axial intensity distributions of HOPS beams with $\eta =+1$. In the case of HOPS beams the axial intensity distributions transform from a central maxima to a twofold symmetric distribution with a dark region in the center surrounded by two intensity lobes that are shifted with $\alpha$.

Row III and IV of Fig. 7 show the axial intensity distributions of right and left-handed HyOPS beams with $I_C=+1$, respectively. Unlike the FPS beams, for HyOPS beams with $\alpha$ the symmetry of the axial intensity distributions varies with $\alpha$. We found that the axial intensity distributions are helicity-dependent. This can be seen by comparing rows III and IV of Fig. 7 for any value of $\alpha$. We find that the helicity inversion of HyOPS beams leads to $180^\circ$ rotation in the axial intensity distribution.

## Conclusion

In conclusion, we numerically demonstrated the focal plane intensity landscapes of optical fields whose polarization states lie on the equator of the relevant Poincaré spheres namely, the fundamental Poincaré sphere, the hybrid order Poincaré sphere, and the higher order Poincaré sphere. We found the focal plane intensity distributions of these optical fields rotate with the rotation angle $\alpha$. The strengths of the component field distributions vary differently with $\alpha$ across these optical fields. We find that only for the HOPS optical fields can the rotation angle $\alpha$ be tuned to achieve equal and non-negligible strengths of longitudinal (*z*) and transverse (*x* and *y*) components at the focal plane. For HOPS optical fields, the maximum intensity of *x* and *y* components varies exactly in the same manner with $\alpha$ at the focal plane; for the HyOPS optical fields, the maximum intensity of *x* and *y* components remains constant independent of $\alpha$. The maximum intensity of the longitudinal (*z*) component of HOPS and HyOPS beams varies with $\alpha$; the range is from 0 to 1 for HOPS beams and 0 to 0.2 for HyOPS beams. For the HyOPS optical fields, the variations of the maximum value of the component intensity distributions with $\alpha$ are helicity-independent. We also found that a helicity inversion of HyOPS beams causes a rotation of $180^\circ$ in the axial intensity distribution. A more uniform intensity profile in multiple dimensions provides a more versatile and stable solution for microstructuring of polarization-sensitive materials, optical micromanipulations, materials micro magnetization, atom optics, interferometry, lithography, high-resolution microscopy, optical trapping, or material machining.

## Data Availability

Data underlying the results presented in this paper are not publicly available at this time but may be obtained from the corresponding author upon reasonable request.
